# Bridging the gap of cardiovascular disease burden in the Americas: a call for action

**DOI:** 10.1016/j.lana.2025.101023

**Published:** 2025-02-14

**Authors:** Daniel Diaz, Diana Z. Velazquez-Valdez, Pavel E. Hernandez-Carreño, Saul A. Beltran-Ontiveros, Edgar L. Gonzalez-Gonzalez, Lina S. Palacio-Mejia

**Affiliations:** a Campus Cuautepec, Universidad Autónoma de la Ciudad de México, Gustavo A. Madero, Ciudad de México, México; b Centro de Investigación en Evaluación y Encuestas, Instituto Nacional de Salud Pública, Cuernavaca, Morelos, México; c Departamento de Biomedicina, Centro de Investigaciones y de Estudios Avanzados, Gustavo A. Madero, Ciudad de México, México; d Centro de Investigación y Docencia en Ciencias de la Salud, Universidad Autónoma de Sinaloa, Culiacán Rosales, Sinaloa, México; e Dirección de Planeación, Instituto Nacional de Salud Pública, Cuernavaca, Morelos, México

In the Americas, cardiovascular diseases (CVD) are a leading cause of mortality and disability.^1^ In this region, the ongoing surge in obesity, diabetes, and sedentary lifestyles has exacerbated the burden of CVD,^2^ which coupled with an inconsistent application of effective prevention measures threatens to worsen this public health issue. The prevailing strategies for addressing CVD have proven inadequate, underscoring the need for a paradigm shift to bridge the gap between evidence and practice. Innovative, scalable, and culturally appropriate solutions are imperative to address the multifaceted risk factors of CVD. To facilitate this timely discussion, a two-part series on Cardiovascular Disease in the Americas was conducted by Joseph, P. et al.,^3^ and Schwalm, J.D. et al.,^4^ In this series, the authors comprehensively examine the burden of CVD in the region, describing its epidemiology, temporal trends, and risk factors.^3^ Additionally, they explore innovative approaches to healthcare and interventions aimed at addressing the loss of health due to CVD.^4^

A paradoxical trend compounds the challenge of reducing the burden of CVD in the Americas. Risk factors such as smoking and cholesterol levels have declined due to effective public health campaigns and government regulations.^5^^,^^6^ However, these gains are overshadowed by the escalating burden of obesity and diabetes.^7^ Furthermore, factors such as poor dietary choices, the consumption of ultra-processed foods, and insufficient physical activity drive the increase in CVD risk.^8^ Additionally, the limited access to healthy environments and sugar-sweetened beverages are contributing factors.^9^ Implementing strategies such as health education campaigns, taxation on unhealthy foods, and regulation of junk food marketing is essential. However, their application is threatened by resistance from industry stakeholders and limited political will. Despite these barriers, several Latin American countries have implemented taxes on sugar-sweetened beverages, though the impact has been heterogeneous due to inconsistent enforcement and geographic reach.

The countries of the Americas must adopt more coordinated and innovative approaches that integrate evidence-based solutions and tackle systemic barriers. For instance, developing effective, inclusive population-wide prevention strategies instead of applying interventions focused on high-risk individuals. Another solution is task shifting, which involves transferring certain healthcare responsibilities from highly trained professionals to non-professional health workers (NPHWs). By training NPHWs to carry out tasks (blood pressure monitoring, medication distribution, and patient education), healthcare systems in low-resource settings can alleviate overworked professionals and bring care closer to underserved populations. Task shifting offers a practical solution to workforce shortages, promoting community-based healthcare. The involvement of family members and peers in CVD management can also be transformative, improving health outcomes. However, to maximize the impact of peers and family support, educational initiatives must be ensured to provide these individuals with the necessary knowledge and tools to support patients effectively.

Digital health technologies represent a promising opportunity to address the geographic and economic barriers to CVD care. In regions where access to healthcare is limited, mobile health tools, telemedicine, and wearable devices could bridge the gap by empowering individuals to monitor their health and adhere to treatment plans. Smartphone applications that track blood pressure or glucose levels, combined with teleconsultations with specialists, can offer a viable solution for patients in remote areas. Nevertheless, challenges such as internet access, affordability, and digital literacy must be addressed to ensure that digital health tools are accessible to all. Governments and stakeholders must prioritize investments in digital infrastructure and ensure that privacy concerns are addressed to foster trust in these technologies.

Simplifying and streamlining clinical guidelines to improve their applicability and effectiveness is essential because sometimes their complexity makes their implementation difficult. Simplifying clinical protocols, such as focusing on key interventions like risk stratification and targeted treatments, can help healthcare providers make better use of limited resources. Moreover, the implementation of fixed-dose combination medications, or polypills (combination of multiple medications into a single pill) could revolutionize CVD care by simplifying treatment regimens and improving patient adherence. While regulatory barriers and limited industry interest present challenges, coordinated efforts from governments, insurers, and pharmaceutical companies could make polypills widely available and affordable.

While tools to address CVD in the Americas already exist, there is a lack of political will and concerted action. To reverse the burden of CVD, the region must scale up successful initiatives and embrace innovative technologies. The time to act is now, as further inaction will lead to high avoidable mortality and a significant economic burden. To this end, governments, healthcare providers, and communities must collaborate to establish a more equitable and sustainable healthcare system that prioritizes the prevention and treatment of CVD. Governments must allocate resources to train NPHWs, procure essential medications, and enhance digital infrastructure. The formation of public-private partnerships is pivotal for aligning the interests of industry, government, and civil society. Finally, public health messaging must be culturally sensitive and tailored to local contexts to ensure that interventions resonate with the target populations.

## Contributors

DD, DZVV, LSPM conceptualization, design, and writing—original draft. All other authors contributed to the writing—review & editing. DD was responsible for the decision to submit the manuscript.

## Declaration of interests

The authors declare no conflict of interest.
